# Analyzing latent categories of stress, anxiety, and depression in medical students: insights into their psychological resilience

**DOI:** 10.3389/fpsyg.2025.1532502

**Published:** 2025-06-13

**Authors:** Huang Lei, Jiang Jiaming, Huang Bin, Yuwen Lyu, Liu Junrong, Lawrence T. Lam, Chen Yufei

**Affiliations:** ^1^Faculty of Medicine of the Macau University of Science and Technology, Macau, Macao SAR, China; ^2^Guangzhou University of Chinese Medicine, Guangzhou, China; ^3^Institute of Humanities and Social Sciences Communications, Guangzhou Medical University, Guangzhou, China

**Keywords:** latent category analysis, psychological resilience, medical students, stress, anxiety, depression

## Abstract

**Objective:**

To explore the potential effects of demographic variables and three factors of psychological resilience, tenacity, strength, and optimism on the stress, anxiety, and depression of medical students, and to provide data support for the refinement of mental health interventions.

**Method:**

A total of 1,099 junior medical students were selected from a certain medical college and surveyed using the Depression Anxiety Stress Scale (DASS-21 Chinese version) and the Connor–Davidson Resilience Scale(CD-RISC Chinese version), as well as a self-designed demographic questionnaire. Data processing was conducted using latent category analysis, contingency table analysis, logistic regression, and other methods.

**Result:**

Three subgroups for stress and depression, and two subgroups for anxiety were obtained. Contingency table analysis results showed that the correlation coefficients between the subgroups and severity were all greater than 0.6. In normal and mild symptomatic populations, latent category analysis fitted the low stress subgroup and the depression subgroup with insufficient motivation. The logistic regression results showed that psychological resilience factors had different effects on the latent categories of stress, anxiety, and depression. Optimism only had a significant predictive effect on the latent category of stress, while for the latent category of anxiety, only strength had a significant predictive effect. The disharmonious or average family atmosphere were common high-risk factors for high stress, high anxiety and high depression subgroups. The experience of living on campus in high school was a unique influencing factor of anxiety among medical students. Male gender and low subjective socio-economic status were unique influencing factors for the high depression group, while left-behind experience was a unique influencing factor for the depression group with insufficient motivation.

**Limitations:**

The explanatory power of cross-sectional studies and non-random sampling is limited, and the universality and misrepresentations of the results need further verification.

**Conclusion:**

There was significant group heterogeneity in the manifestations of stress, anxiety, and depression among medical students, and the behavioral response patterns of subgroups with latent categories exhibited cross group characteristics when grouped by the norm. The impact of tenacity, strength, and optimism on subgroups of stress, anxiety, and depression varied. Future research should integrate different research paradigms, deepen understanding, and provide more targeted evidence support for psychological education and intervention programs for medical students.

## 1 Background

Based on the significant negative impact on personal growth and academic development, as well as the predictive role in individual psychological and behavioral problems (Nikolic et al., 2023; Lew et al., 2019), the stress, anxiety, and depression levels of college students have received widespread attention from researchers. Yu and Huang (2023) meta-analysis of the detection rate of mental health problems among Chinese students showed that the detection rate of depression and anxiety among Chinese university students was 20.8 and 13.7%, respectively. The survey results of the Chinese National Mental Health Development Report (2021–2022) showed that the detection rate of depression risk in the 18–24 age group was as high as 24.1%, significantly higher than other age groups, with young people being a high-risk group for depression (Fu and Zhang, 2023). Among them, medical college students, due to the high requirements of society for their future career “morality” and “skills,” faced academic assessment pressure of theoretical and practical long-term education during their education and training process. At the same time, the special nature of doctor-patient relationships requires them to have additional exercise in interpersonal communication, as well as other pressures they may face. Compared with other universities and professional college students, Li et al. (2022) meta-analysis of the prevalence of depression and anxiety symptoms among global college students showed that the prevalence of depression symptoms among medical college students has increased by 39.4% (95% CI: 29.3–49.6%), and the prevalence of anxiety symptoms has increased by 47.1% (95% CI: 35.1–59.1%). The anxiety and depression risks faced by medical college students have been confirmed worldwide to be generally high. The meta-analysis study by Mao et al. (2019) showed that the average prevalence of depression and anxiety among Chinese medical students was 32.74 and 27.22%, respectively. However, for lower grade medical students, especially freshmen, due to the changing identity status they face during this transition period, changes in interpersonal relationships, regional culture, lifestyle habits, and academic performance require them to expend a great deal of energy to adapt. Relevant studies have shown that lower grade medical students have more prominent levels of stress perception, anxiety, and depression (Chen et al., 2024; Yang et al., 2023; Yang et al., 2024; Li et al., 2024).

However, the environment is not the only factor leading to poor mental health among medical students. Psychological resilience, as a positive internal factor, plays an important role in protecting and promoting individual mental health. Psychological resilience refers to a psychological phenomenon in which an individual experiences severe stress/adversity, but their physical and mental condition is not damaged, and instead becomes stronger and stronger (Xi et al., 2008). The phenomenon of psychological resilience reflects the diversity of individual development and a positive perspective in the face of pressure or adversity. Studies have shown that psychological resilience is not an isolated phenomenon, but a common developmental tendency (Shevlin et al., 2023; Bonanno et al., 2024). Cross sectional health surveys and meta-analysis studies from clinical and non-clinical samples have shown a direct correlation between higher levels of psychological resilience and fewer mental health problems (Imran et al., 2024; Weitzel et al., 2022; Yu et al., 2024). The results of cohort studies have shown that psychological resilience can predict the trajectory of individuals’ negative and positive emotions (Zhang et al., 2021).

However, many studies have also pointed out that individuals’ responses to stress, their manifestations of anxiety and depression symptoms, and their responses to interventions vary to varying degrees, and the objective fact of heterogeneity within the group should be respected (Fulambarkar et al., 2023; Maity et al., 2024). At present, research on group differentiation of stress, anxiety, and depression symptoms among college students (including medical students) is focused on variable centered cross-sectional studies, mainly involving socio-demographic variables and educational background information, investigating the effects of variables such as gender, age, partner relationships, personal educational experience, family factors, interpersonal communication factors, and communication with teachers (Nayak and Sahu, 2022; Fauzi et al., 2021; Rabby et al., 2023; Gao et al., 2020; Ramón-Arbués et al., 2020). Considering the heavy burden of stress, anxiety, and depression on public health and family socio-economic aspects of college students, especially medical students, effective and meaningful categories are further explored. Identification is of great significance for etiological research, education and clinical intervention practices, as well as for education management.

Latent Category Analysis (LCA) is a core statistical tool of the human centered research paradigm used to identify potential subgroups in data, widely used in fields such as psychology, sociology, medicine, and market research. Its core idea is to assume that the heterogeneity of observed data is driven by several “latent categories” that are not directly observed, and each category has a unique response pattern Ulbricht et al. (2018) conducted a systematic evaluation study that showed that the use of LCA to explore the subtypes of depression in clinical samples was quite common, while in non clinical samples, Gordon Parker et al. (2019) used LCA to identify and differentiate depressive disorders. Liu et al. (2021) studied the anxiety and depression potential categories of Chinese college students in the context of the COVID-19, and obtained three potential categories, including the poor mental health group with serious depression and anxiety symptoms, the mild symptom group with mild emotional distress as the main symptom, and the low symptom group with better mental health.

This study adopted a people-centered LCA technique to investigate the heterogeneity of stress, anxiety, and depression among lower grade medical students, and examined the effects of demographic variables and psychological resilience levels on subgroups of stress, anxiety, and depression latent categories, in order to provide data support for the refinement of mental health interventions.

## 2 Objects and methods

### 2.1 Object

In March 2024, a convenience sampling method was used to survey 1,219 lower grade medical students (average age 19.4 years, standard deviation 2.08) at a medical college. By removing questionnaires such as invalid answers, contradictory answers, and missing key information, 1,099 valid questionnaires (90.16%) were actually collected. All survey subjects had informed consent for this study, which has been approved by the Ethics Committee of Guangdong Provincial Hospital of Chinese Medicine (Ethics Review Approval No.: ZE2024-054-01).

### 2.2 Methods

#### 2.2.1 Collection of demographic data

We used a self-designed questionnaire to collect demographic data from participants, including gender, family atmosphere (FA), one-child status (OCS), left-behind experience (LBE), whether they live on campus in high school (WLC), and whether they are student leaders (WSL). The subjective socioeconomic status (SSS) data of the subjects and their families were obtained using the MacArthur Scale for Subjective Socioeconomic Status (Liu and Wang, 2020).

#### 2.2.2 Depression anxiety stress scale (Chinese version)

The Chinese version of the Depression Anxiety Stress Scale (DASS-21), revised by Gong Xu et al. in 2010, was used. The scale consists of 21 items and includes three subscales: depression, anxiety, and stress. Each subscale contains 7 items and is scored on a scale of 0–3, with 0 indicating non conformity, 1 indicating occasional conformity, 2 indicating frequent conformity, and 3 indicating always conformity. This scale has been validated among Chinese university students (Gong et al., 2010) and also among freshmen (Cao et al., 2023). The internal consistency coefficients of the three subscales are: 0.77, 0.79, and 0.76; the internal consistency coefficient of the total scale is 0.89; the construction reliability of the three scales is 0.72, 0.80, and 0.76.

#### 2.2.3 Connor Davidson resilience scale (Chinese version)

The Chinese version of the Connor Davidson Resilience Scale, translated and revised by Yu and Zhang (2007), contains 25 items consisting of three dimensions: tenacity, strength, and optimism. It uses a Likert 5-point scoring system, ranging from 0 (strongly disagree) to 4(strongly agree). The higher the score on the scale, the better the psychological resilience of the research subjects is. The Chinese version has been widely used among different groups in China, with an internal consistency coefficient of 0.91.

### 2.3 Statistical analysis

The Mplus 7.0 software was used for latent category analysis (LCA). The evaluation indicators of the latent category model include AIC: Akaike Information Criterion; BIC: Bayesian Information Criterion; ABIC: BIC for sample calibration; BLRT: Bootstrap Likelihood Ratio Test (K-1 vs. K classes); VLMR: Vuong Lo Mendel Rubin Likelihood Ratio Test (K-1 vs. K classes); Entropy: the smaller the AIC, BIC, and ABIC values, the better the model fit, when the Entropy≥0.8,the classification accuracy exceeds 90%; if the *p*-values of BLRT and VLMR are less than 0.05, it indicates that the k-category model is superior to the k-1 category model. Referring to the study by Sinha et al. (2021), it is determined that the proportion of people in each category should not be less than 10%.

Since DASS-21 measures three indicators of stress, anxiety, and depression, rather than three dimensions of a single indicator, the LCA method is used to classify the stress, anxiety, and depression status of medical students separately. The DASS-21 entry is assigned a score of 0–3. Considering that in this sample, the response of “Applied to me very much, or most of the time” with a score of 3 for each entry is less than 5%, and considering that LCA is sensitive to outlier, the responses with scores of 2 and 3 will be merged and classified into three categories for latent category analysis.

SPSS 26.0 software was used for data processing and statistical analysis. Categorical variables were represented by frequency and composition ratio, and continuous variables were represented by mean and standard deviation. The inter group differences of categorical variables were compared using contingency table analysis, and logistic regression analysis was used to explore the influencing factors of medical students’ stress, anxiety, and depression.

## 3 Results

### 3.1 Potential category analysis of stress, anxiety, and depression in medical students

The fitting results show that when the stress level of medical students is at k = 3, the Entropy value is 0.872, and the *p*-values corresponding to BLRT and VLMR are both less than 0.01. Moreover, the downward trend of AIC, BIC, and ABIC slows down, and the slope gradually approaches 0. When the anxiety level of medical students is at k = 2, the Entropy value is 0.870, and the p-values corresponding to BLRT and VLMR are both less than 0.01, and the proportion of people in each category is not less than 10%. When the depression level of medical students is at k = 3, the Entropy value is 0.812, and the p-values corresponding to BLRT and VLMR are both less than 0.01. Moreover, the downward trend of AIC, BIC, and ABIC slows down and gradually approaches 0. Taking into account the simplicity of the model and the research objectives, this study retains the 3-category stress and depression latent category model and the 2-category anxiety latent category model, as shown in Tables 1–3.

**Table 1:** Potential category model fitting results of medical students’ stress levels.

Category	AIC	BIC	ABIC	Entropy	BLRT *p*	VLMR *p*	Category probability (%)
1C	14724.811	14794.841	14750.374	—	—	—	—
2C	12404.792	12549.855	12457.744	0.880	<0.001	<0.001	47.6/52.4
3C*	11878.444	12098.539	11958.785	0.872	<0.001	<0.001	10.3/44.9/44.8
4C	11707.231	12002.358	11814.961	0.798	<0.001	<0.01	27.0/11.4/35.3/26.3
5C	11653.064	12023.224	11788.183	0.755	0.207	0.042	10.7/23.5/16.1/24.7/25.0

— Indicates no such data; *Best fit model.

**Table 2:** Potential category model fitting results of anxiety levels in medical students.

Category	AIC	BIC	ABIC	Entropy	BLRT *p*	VLMR *p*	Category probability (%)
1C	13142.916	13212.946	13168.479	—	—	—	—
2C*	11130.173	11275.235	11183.124	0.870	<0.001	<0.001	36.7/63.3
3C	10801.176	11021.271	10881.517	0.871	<0.001	<0.001	7.8/31.1/60.1
4C	10610.564	10905.691	10718.293	0.789	<0.001	0.0011	38.7/16.7/36.6/8.0
5C	10557.098	10927.257	10692.216	0.746	0.056	0.297	18.0/11.6/31.7/6.1/32.7

— Indicates no such data; *Best fit model.

**Table 3:** Potential category model fitting results of depression levels in medical students.

Category	AIC	BIC	ABIC	Entropy	BLRT *p*	VLMR *p*	Category probability (%)
1C	12818.525	12888.556	12844.088	—	—	—	—
2C	10570.001	10715.064	10622.953	0.865	<0.001	<0.001	35.3/64.7
3C^*^	10159.837	10379.932	10240.178	0.812	<0.001	<0.001	19.3/36.0/44.7
4C	9924.245	10219.372	10031.974	0.834	<0.001	0.0096	34.7/10.9/43.7/10.7
5C	10382.045	10927.280	10581.070	0.857	<0.001	0.8357	2.3/7.9/35.6/10.5/43.8

— Indicates no such data; *Best fit model.

### 3.2 Characteristics and naming of various categories of stress, anxiety, and depression in medical students

**Figure 1:**
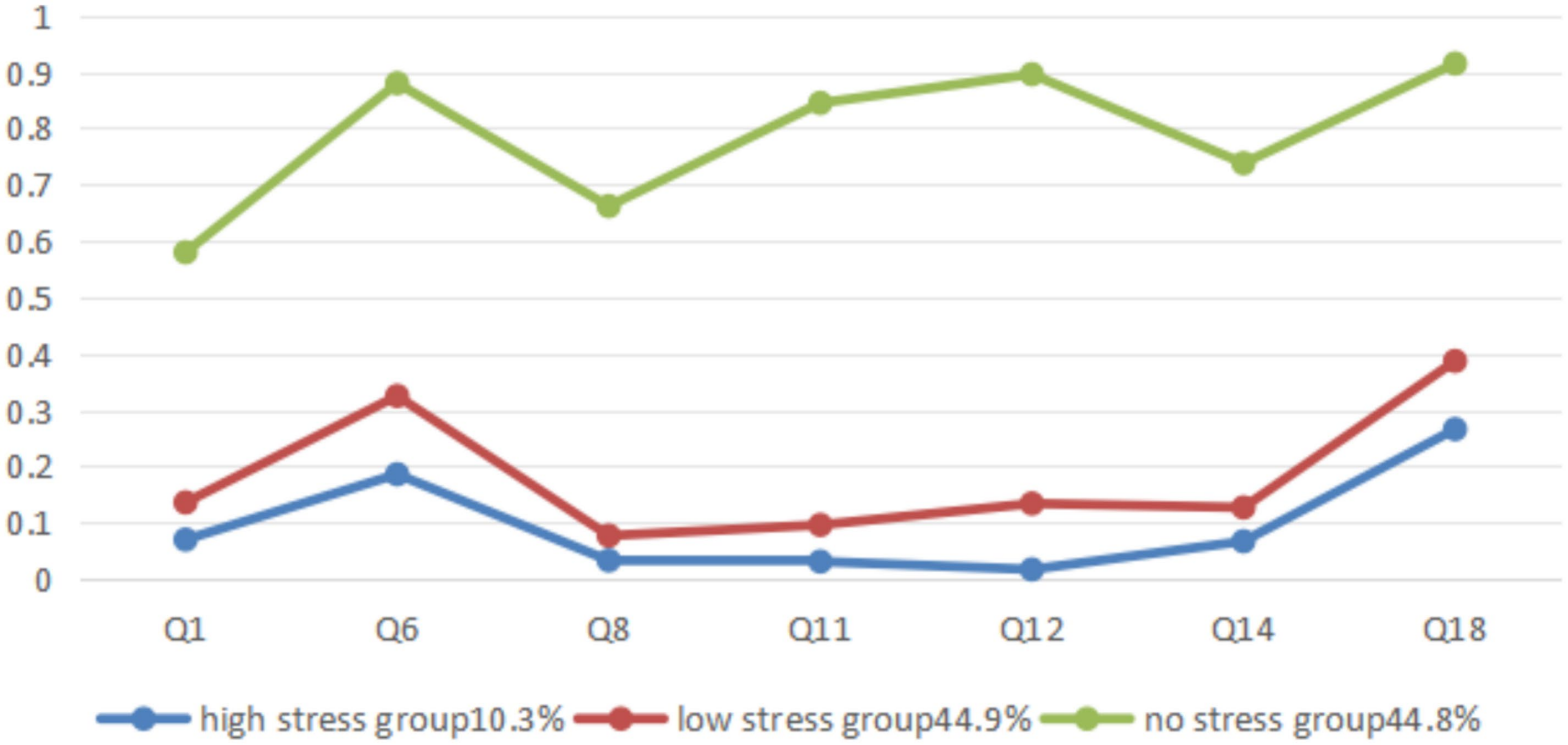
Potential category grouping and conditional probability diagram of stress level for medical students.

In this study, the conditional probability was set as the response probability for the option “Did not apply to me at all” in each entry. Based on the conditional probability level, the subgroups of the three latent categories of stress are named as high stress group, low stress group, and no stress group. The stress conditional probability plot shows that the high and low stress groups generally have low probability responses to the stress 7 items in DASS, but have high response probabilities on the Q6 (I tended to over react to situations) and Q18 (I felt I was a rat touch) items. The average scores of Q6 and Q18 were calculated, and the results show that the mean of Q6 was 0.53 and the mean of Q18 was 0.47, reflecting the lower allergic reactions and tactile anger in this sample group under stress conditions (Figure 1).

**Figure 2:**
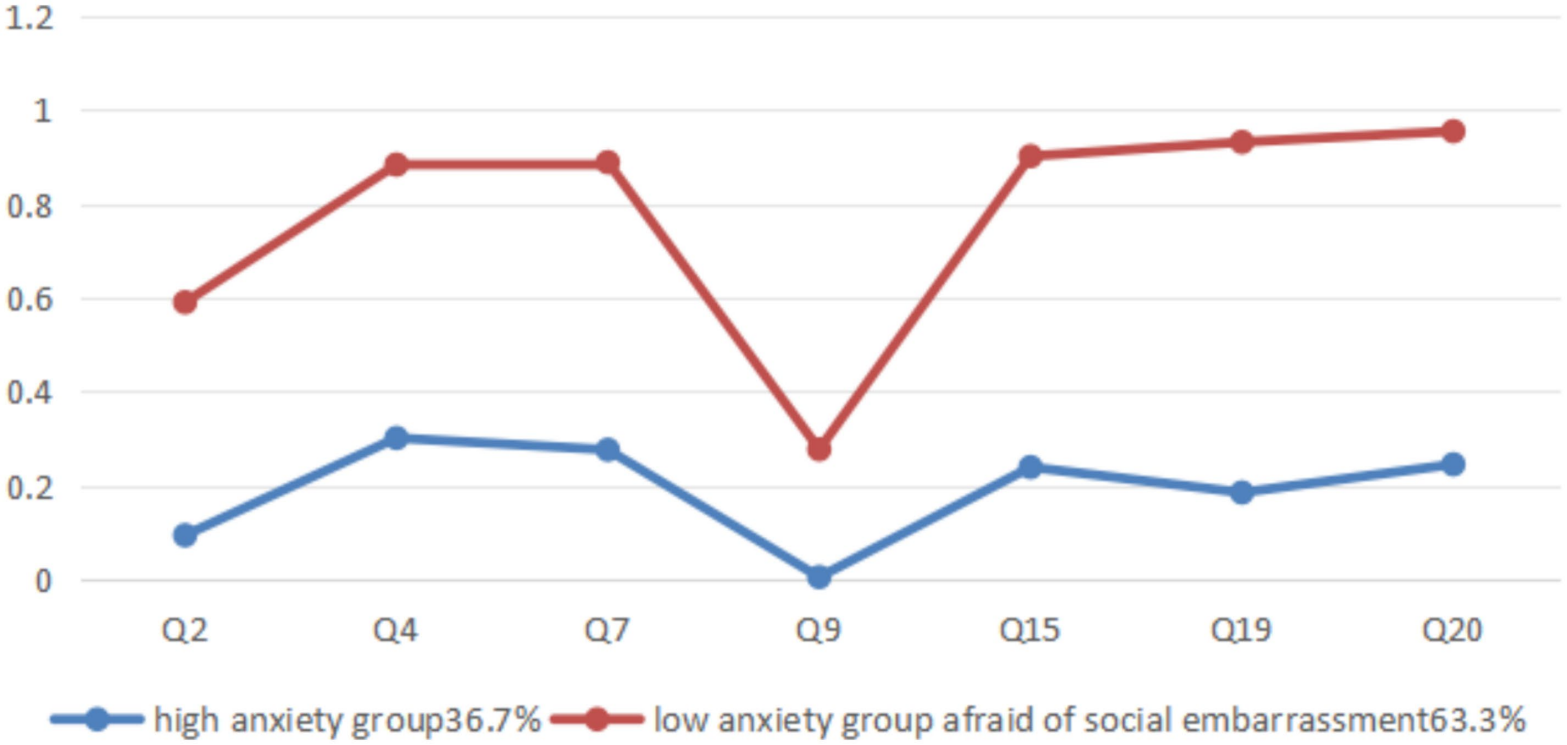
Subclass grouping and conditional probability diagram of anxiety levels among medical students.

The anxiety conditional probability plot shows that the high anxiety group exhibits a generally low probability response to the seven items of stress in DASS, but the response probability on Q9 (I was worried about situations in which I might panic and make a fool of myself) is lower, and the low anxiety group has a higher response probability on all six items, but shows a significant low response to Q9, which is similar to the high anxiety group. Therefore, the low anxiety group is named as “low anxiety group afraid of social embarrassment (LAGASE).” The average Q9 of the entire sample is 1.31, reflecting the general anxiety of the sample group toward social embarrassment (Figure 2).

**Figure 3:**
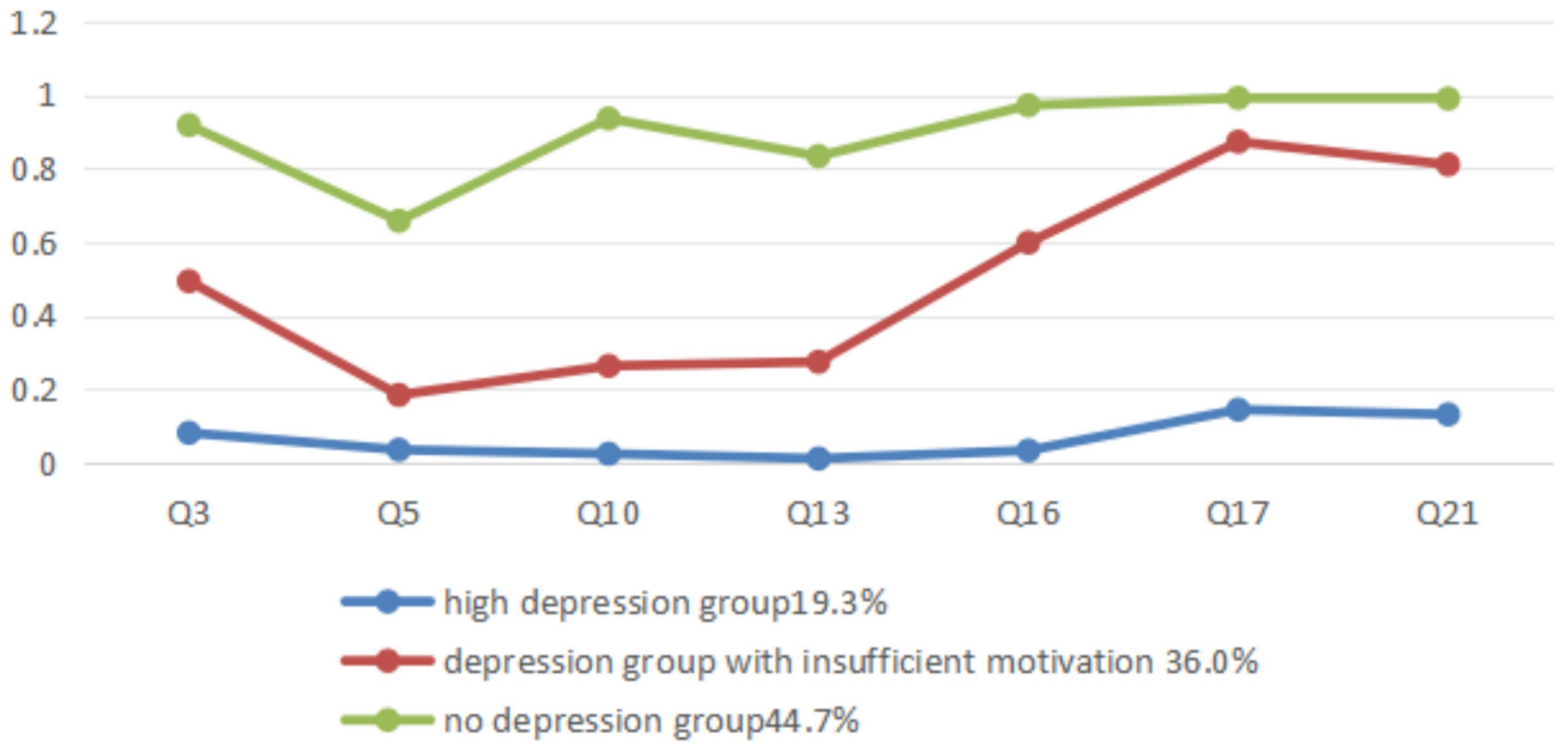
Subclass grouping and conditional probability diagram of depression levels among medical students.

The depression conditional probability plot shows that the three latent categories have different response patterns to the seven items of depression. The high depression group shows a low response probability on all items, while the non-depression group has a lower response probability in Q5 (I found it difficult to work up the initiative to do things) compared to the high response probability on the other six items. Between the high depression and non-depression groups, an intermediate group is classified, which shows a high response probability to Q17 (I felt that I was worth much as a person) and Q21 (I felt that life was meaningless), and a low response probability to Q5 (I found it difficult to work up the initiative to do things), Q10 (I felt that I had nothing to look forward to) and Q13 (I felt down hearted and blue). The statistical results show that Q5 scoring 0.81, Q10 scoring 0.61, Q13 scoring 0.63, Q17 scoring 0.27, and Q21 scoring 0.3. It indicates that the middle group generally reflects a lack of motivation, depression, and frustration, but rarely experiences self-deprecation and a sense of meaninglessness in life, hence the name “depression group with insufficient motivation (DGIM; Figure 3).

### 3.3 Comparison of subgroup and severity grouping of stress, anxiety, and depression in medical students

The stress detection rate, anxiety detection rate, and depression detection rate in the sample of this study were 21.7, 46.4, and 31.7%, respectively. According to the norm classification standard, the scores of stress, anxiety, and depression in the sample were classified into four groups: normal, mild, moderate, severe and above. Corresponding to the stress latent category subgroup, anxiety latent category subgroup, and depression latent category subgroup, cross comparison was conducted in a contingency table. The results showed that there was a considerable degree of consistency between the latent category subgroup and the severity subgroup based on the norm (the contingency coefficients were all greater than 0.6), but the differences between the two were also significant. Please refer to Tables 4–6 for details.

**Table 4:** Contingency table analysis between stress latent category grouping and stress level grouping based on norm.

Latent category grouping		Grouping of stress levels based on norm				Total
		Normal	Mild	Moderate	Severe and above	
Subgroups	High stress group	3	13	55	42	113
	Low stress group	375	104	14	0	493
	No stress group	493	0	0	0	493
Total		871	117	69	42	1,099
Pearson chi square		944.378**				
Contingency coefficient		0.680**				

*p<0.05, **p<0.01, ***p<0.001.

**Table 5:** Contingency table analysis between anxiety latent category grouping and norm based anxiety level grouping.

Latent category grouping		Anxiety level grouping based on norm				Total
		Normal	Mild	Moderate	Severe and above	
Subgroups	High anxiety group	0	66	184	153	403
	LAGASE	589	101	6	0	696
Total		589	167	190	153	1,099
Pearson chi square		902.098**				
Contingency coefficient		0.671**				

*p<0.05, **p<0.01, ***p<0.001.

**Table 6:** Contingency table analysis between depression latent category grouping and norm based depression severity grouping.

Latent category grouping		Depression level grouping based on norm				Total
		Normal	Mild	Moderate	Severe and above	
Subgroups	High depression group	0	3	170	39	212
	DGIM	262	127	7	0	396
	No depression group	489	1	1	0	491
Total		751	131	178	39	1,099
Pearson chi square		1254.032**				
Contingency coefficient		0.730**				

*p<0.05, **p<0.01, ***p<0.001.

The latent category of no pressure group is all distributed in the normal pressure group, the vast majority of the latent category of high pressure group is distributed in the mild, moderate, severe and above pressure level groups, mainly in the moderate, severe and above levels, and the latent category of low pressure is distributed in the normal, mild and moderate pressure groups, mainly in the normal and mild pressure groups. The LAGASE included all normal anxiety samples, and also included mild and moderate anxiety samples based on response patterns, with mild anxiety samples being the main group. The latent high anxiety group is distributed in the mild, moderate, severe and above anxiety levels, with the majority of samples being moderate, severe and above anxiety levels. The latent high depression group is distributed in the mild, moderate, severe and above degree depression groups, with moderate, severe and above degree depression samples being the main ones. The vast majority of the latent non depression group is distributed in the normal depression level group, while the DGIM is mainly distributed in the normal depression group and the mild depression group.

### 3.4 Factors influencing subgroups of stress, anxiety, and depression in medical students

#### 3.4.1 Single factor analysis of subgroups of underlying categories of stress, anxiety, and depression affecting medical students

Comparing the differences in demographic variables such as gender among the latent categories of stress, anxiety, and depression, the results showed that the subgroups of the latent categories of stress had statistically significant differences in SSS and FA, while the subgroups of the latent categories of anxiety had statistically significant differences in SSS, LBE, WLC, and FA. The subgroups of the latent categories of depression had statistically significant differences in gender, SSS, LBE, WSL, and FA. See Table 7 for details.

**Table 7:** Univariate analysis of potential factors influencing stress, anxiety, and depression in medical students.

Demographic variables		HPG	LPG	NPG	Χ^2^ value	HAG	LAGASE	Χ^2^ value	HDG	DGIM	NDG	Χ^2^ value
Gender	Male	44	161	154	2.48	136	267	0.34	85	122	152	6.59*
	Female	69	332	339		223	473		127	274	449	
SSS	Low	22	50	31	28.93***	55	48	23.59***	38	39	26	42.60***
	Medium	75	348	328		285	468		1,448	275	328	
	High	16	95	134		65	180		26	82	137	
OCS	Yes	35	126	137	1.57	106	192	0.21	63	94	141	3.65
	No	78	367	356		297	504		149	302	350	
LBE	Yes	20	103	77	4.63	88	112	5.66*	44	86	70	9.35**
	No	93	390	416		315	584		168	310	421	
WSL	Yes	47	189	221	4.27	154	303	2.98	74	153	230	10.90**
	No	66	304	272		249	393		138	243	261	
WLC	Yes	104	448	444	0.49	378	618	7.56**	197	364	435	4.48
	No	9	45	49		25	78		15	32	56	
FA	Disharmonious	12	17	15	42.36***	28	16	38.30***	19	11	14	52.45***
	Average	41	140	88		129	140		73	114	82	
	Harmonious	60	336	390		246	540		120	271	395	

*p<0.05, **p<0.01, ***p<0.001. HSG-high stress group, LSG-high stress group, NSG-no stress group, HAG-high anxiety group, LAGASE-low anxiety group afraid of social embarrassment, HDG-high depression group, DGIM-depression group with insufficient motivation, NDG-no depression group.

#### 3.4.2 Multivariate logistic regression analysis of factors influencing subgroup factors of stress, anxiety, and depression in medical students

Using subgroups of stress, anxiety, and depression as dependent variables, demographic variables with statistical significance in univariate analysis as independent variables, and psychological resilience as covariate, a disordered multiple logistic regression analysis was conducted. The results showed that tenacity, strength, and optimism were the influencing factors of the high and low stress groups, while SSS , disharmony and average in FA only had significant predictive effects on the high stress group; strength, WLC, and disharmony and average in FA were influencing factors for the high anxiety group; tenacity, strength, male gender, LBE, low SSS, and average in FA were the influencing factors of the high depression group, while tenacity, strength, LBE, and FA were generally the influencing factors of the DGIM. Please refer to Tables 8–10 for details.

**Table 8:** Multivariate logistic regression analysis of factors influencing subgroups of stress potential categories among medical students.

Independent/covariate variables		B	S. E.	Wald	*p*-value	OR	95%CI	
High stress group	Tenacity	−0.08	0.03	7.52	0.01	0.93	0.88	0.98
	Strength	−0.13	0.05	6.49	0.01	0.88	0.80	0.97
	Optimistic	−0.14	0.07	4.23	0.04	0.87	0.77	0.99
	[SSS = low]	0.83	0.43	3.71	0.05	2.28	0.99	5.29
	[SSS = medium]	0.12	0.31	0.15	0.70	1.13	0.61	2.09
	[FA = disharmony]	0.98	0.48	4.26	0.04	2.66	1.05	6.75
	[FA = average]	0.58	0.26	5.13	0.02	1.79	1.08	2.95
Low stress group	Tenacity	−0.04	0.02	5.91	0.02	0.96	0.93	0.99
	Strength	−0.09	0.03	7.40	0.01	0.92	0.86	0.98
	Optimistic	−0.10	0.04	6.26	0.01	0.90	0.83	0.98
	[SSS = low]	0.33	0.29	1.28	0.26	1.39	0.79	2.44
	[SSS = medium]	0.08	0.17	0.25	0.62	1.09	0.78	1.51
	[FA = disharmony]	−0.08	0.39	0.04	0.85	0.93	0.43	2.00
	[FA = average]	0.29	0.17	2.98	0.09	1.34	0.96	1.86

Refer to the non-stress group.

**Table 9:** Binary logistic regression analysis of factors influencing the subgroup of anxiety in medical students.

Independent/covariate variables	B	S. E.	Wald	*p*-value	OR	95%CI	
[SSS = low]	−0.48	0.27	3.14	0.08	0.62	0.36	1.05
[SSS = medium]	−0.18	0.18	0.97	0.32	0.84	0.59	1.19
[LBE = yes]	−0.28	0.17	2.57	0.11	0.76	0.54	1.06
[FA = disharmony]	−1.03	0.36	8.32	0.00	0.36	0.18	0.72
[FA = average]	−0.33	0.16	4.32	0.04	0.72	0.53	0.98
[WLC = yes]	−0.55	0.26	4.39	0.04	0.58	0.35	0.97
Tenacity	0.02	0.02	0.84	0.36	1.02	0.98	1.05
Strength	0.14	0.03	18.41	0.00	1.15	1.08	1.22
Optimistic	0.07	0.04	2.74	0.10	1.07	0.99	1.16

Taking the high anxiety subgroup as a reference.

**Table 10:** Multivariate logistic regression analysis of influencing factors on subgroups of depression in medical students.

Independent/covariate variables		B	S. E.	Wald	*p*-value	OR	95%CI	
High depression group	Tenacity	−0.05	0.02	4.91	0.03	0.95	0.90	0.99
	Strength	−0.28	0.05	39.14	0.00	0.75	0.69	0.82
	Optimistic	−0.05	0.06	0.78	0.38	0.95	0.85	1.06
	[SSS = low]	1.05	0.39	7.19	0.01	2.87	1.33	6.18
	[SSS = medium]	0.27	0.27	1.00	0.32	1.31	0.77	2.21
	[FA = disharmony]	0.87	0.45	3.69	0.06	2.39	0.98	5.81
	[FA = average]	0.54	0.23	5.77	0.02	1.72	1.10	2.67
	[Gender = male]	0.57	0.21	7.72	0.01	1.77	1.18	2.64
	[LBE = yes]	0.42	0.25	2.86	0.09	1.52	0.94	2.47
	[WSL = yes]	−0.02	0.20	0.01	0.91	0.98	0.66	1.45
DGIM	Tenacity	−0.05	0.02	7.25	0.01	0.95	0.92	0.99
	Strength	−0.12	0.04	12.01	0.00	0.89	0.83	0.95
	Optimistic	−0.02	0.04	0.29	0.59	0.98	0.90	1.06
	[SSS = low]	0.42	0.32	1.76	0.18	1.52	0.82	2.81
	[SSS = medium]	−0.03	0.18	0.02	0.88	0.97	0.69	1.38
	[FA = disharmony]	−0.14	0.44	0.10	0.76	0.87	0.37	2.07
	[FA = average]	0.40	0.18	5.00	0.03	1.50	1.05	2.13
	[Gender = male]	0.18	0.16	1.21	0.27	1.19	0.87	1.64
	[LBE = yes]	0.45	0.19	5.39	0.02	1.56	1.07	2.28
	[WSL = yes]	−0.13	0.15	0.74	0.39	0.88	0.66	1.18

The no depression group is used as a reference.

## 4 Discussion

### 4.1 There is group heterogeneity in the manifestations of stress, anxiety, and depression among medical students

Based on the DASS-21 norm, the anxiety detection rate of medical students in this study was 46.4%, and the depression detection rate was 31.7%, which is similar to previous research results and significantly higher than the average level of Chinese university students.

Among the three subgroups of stress potential categories, the high stress potential category group and the non-stress potential category group have a strong correspondence with the severity grouping based on the norm. At the same time, a low stress group was identified in the main population with normal levels. This subgroup exhibited a series of tense behavioral patterns, but had low allergic reactions and low feelings of anger. On the seven items of anxiety, both high and low anxiety subgroups showed concerns and fears about making a fool of themselves in social situations. Among the three subgroups of depression, the high depression group and the non-depression group have a strong correspondence with the severity grouping based on the norm. At the same time, a depression group with insufficient motivation was identified in the population with normal levels. This subgroup exhibited behavioral patterns such as insufficient motivation, depression, and frustration, but rarely had self-deprecation and a sense of meaninglessness in life. Compared with the severity grouping of the norm, latent category analysis captures some special behavioral patterns of stress, anxiety, and depression in the medical student population, and these patterns have cross group characteristics between groups grouped by severity. It can be considered that latent category analysis based on response patterns and conditional probability has its unique contribution to distinguishing the heterogeneity of stress, anxiety, and depression in the medical student population, and can be combined with norm classification to provide more targeted intervention guidelines.

### 4.2 Demographic factors influencing subgroups of stress, anxiety, and depression in medical students

It is generally believed that the incidence rate of depression in women is higher than that in men. The results of this study showed that compared with the non-depression group, men were significant predictors of high depression (OR: 1.77, *p*: 0.01, 95% CI: 1.18–2.64). Without distinguishing majors, the meta-analysis study by Yu and Huang (2023) showed no statistically significant difference in depression scores between men and women, while a meta-analysis study of medical college students showed that men were more depressed than women (OR: 1.17, 95% CI: 1.10–1.24; Mao et al., 2019). Based on the social factor hypothesis, relevant studies suggest that Chinese society generally has higher expectations for men’s economic ability and family social responsibility, and the pressure of long-term education and uncertainty about future employment faced by medical students may be one of the important reasons for the higher depression detection rate among men than women (Chen et al., 2013; Pan et al., 2016). Considering that gender does not have a significant impact on the depression group with low motivation, unlike the high depression group, which mainly has moderate to severe depression, and the depression group with low motivation, which mainly consists of normal and mildly depressed individuals, the above explanation should also consider the susceptibility factors of individuals with high depression based on the Comprehensive Quality Stress Model (Parker and Brotchie, 2010).

The results of meta-analysis showed that left-behind children exhibited higher rates of depression and anxiety compared to their peers (Cheng and Sun, 2015). For medical college students, this study found that previous experiences of left behind children were a significant predictor of depression with low motivation, reflecting the long-term impact of left-behind experiences on individual psychology. Left-behind (separation from father/mother) means the loss of actual or symbolic attachment objects. From the perspective of attachment theory, left behind is a disturbance and destruction of secure attachment relationships, and insecure attachment is considered an important risk factor for mental health problems and a core element in the occurrence and development trajectory of mental disorders. The meta-analysis results of Herstell et al. (2021) showed that insecure attachment is a cross diagnostic common risk factor for bipolar disorder, depression, and schizophrenia spectrum disorders.

Since middle school, many Chinese students have started to leave their families and move into schools. In this study, 90.6% of medical students had experience living on campus, reflecting the prevalence of living on campus. Compared to the low anxiety group, the experience of living on campus was a significant predictor for the high anxiety group. Living on campus means living and learning with others. From the common concern and fear of social embarrassment exhibited by medical students, it can be considered that in a cultural environment that emphasizes interpersonal harmony with others, living on campus increases an individual’s social chances in the group, combined with the psychological pressure brought by the Chinese expectation of “building good relationships” with others, and jointly promotes anxiety about social embarrassment.

In this study, low subjective socioeconomic status was a common predictor for the high stress group, high anxiety group, and high depression group, similar to the results of Präg et al. (2016) based on data from 29 countries, which confirmed a robust association between subjective socioeconomic status (SSS) and self-rated health (SRH) and psychological well-being outcomes. SSS showed a significant negative correlation with psychological symptoms of SRH and a significant positive correlation with psychological well-being, and this result was universal across all study countries.

Logistic regression revealed the importance of family atmosphere as a factor. Compared to harmonious family atmosphere, disharmony or average in family atmosphere can significantly increase the chances of medical students being classified into high stress group, high anxiety group, and high depression group. According to Olson (2011) understanding of family atmosphere, harmonious family atmosphere is defined as a balance of cohesion, flexibility, and communication quality. A systematic evaluation of the relationship between family atmosphere and adolescent adaptability shows a significant correlation between family atmosphere and adolescent adaptive behavior (Kurock et al., 2022). According to Bronfenbrenner (1979) ecosystem theory, the family as a microsystem has a direct impact on individual development, and supportive family environments can enhance individuals’ ability to cope with stress. Cross cultural studies have shown that family influence is more prominent in collectivist cultures (Asia; He et al., 2023).

### 4.3 Different effects of psychological resilience factors on subgroups of underlying stress, anxiety, and depression in medical students

The systematic review and meta-analysis conducted by Luo et al. (2025) on the correlation between psychological resilience and mental health in adolescents and youth groups showed that the correlation coefficient between psychological resilience and negative indicators of mental health was −0.391 (95% CI: −0.469, −0.308, *p* < 0.001), and the correlation coefficient with positive indicators of mental health was 0.499 (95% CI: 0.400, 0.586, *p* < 0.001). This study refined the relationship between the three factors of psychological resilience (tenacity, strength, and optimism) and stress, anxiety, and depression. The results showed that psychological resilience factors have different effects on the subgroups of medical students in the latent categories of stress, anxiety, and depression.

The optimistic physical and mental benefits have been supported by a wealth of evidence in previous studies (Scheier and Carver, 2018; Rasmussen et al., 2009), and in this study, the optimistic factor has a significant predictive effect on stress groups. Bonanno et al. (2024) defines optimism as a motivation and belief that accepting challenges is valuable, believing that they can and ultimately will effectively move toward a positive future. Based on this, optimists provide the necessary motivational push, handle cognitive and emotional events in adversity, and initiate behavioral adjustments they may need. Carver and Scheier (2017) used a healthy biological behavior model to explain the mechanism of the relationship between optimism and individual physical and mental health. They believe that there are three important pathways of action. The first is the behavioral pathway mediated by differential coping strategies. The experimental research results of Wang and Wang (2023) showed that individuals facing different levels of negative situations are more inclined to choose positive reappraisal strategies compared to pessimists, and can flexibly choose multiple cognitive reappraisal strategies to regulate negative emotions. There is considerable evidence to suggest that optimism is associated with the use of positive, method oriented coping strategies, which directly address the problems faced or change perceptions of stressors in a positive way, thereby exerting adaptive functions (Carver and Scheier, 2017). The second is the physiological pathway of stress response. Optimism is related to the immune response to stress response. In the face of stressful situations, optimists tend to persist in overcoming stressors, which is reflected at the physiological level, that is, sustained physiological stress response and cell-mediated immune reduction. Researchers believe that although this phenomenon may lead to an increase in stress levels or even disorder in the short term, this strategy may have long-term benefits in ultimately solving stressors (Segerstrom, 2005). The third is social support and social integration pathways. Researches have shown that optimism promotes more beneficial social relationships and networks (Smith et al., 2013; Andersson, 2012). The three approaches reflect the important impact of optimism on individuals’ perception of stress and stress management in the face of adversity or stressors from different perspectives.

Yu and Zhang (2007) have argued that tenacity described a person’s perseverance, calmness, agility, and sense of control in the face of difficulties and challenges, while strength focused on an individual’s ability to recover and become stronger after experiencing setbacks.

The results showed that tenacity and strength were significant predictive factors for the high depression group, while strength was a significant predictive factor for the high anxiety group. The resource conservation theory proposed by Hobfoll (1989, 2002) provided a reference explanatory model for this impact. Hobfoll (1989, 2002) resource conservation theory suggested that in stressful situations, a person’s willingness to act and subsequent behavioral activities were regulated by two orientations: resource gain orientation and resource loss orientation. Hobfoll defines resources as personal characteristics, objects, conditions, or energy that were valued by individuals, or as means, personal characteristics, conditions, or energy to achieve these objects. The study by Bouckenooghe et al. (2019) considered tenacity and strength as loss oriented resources. Based on the framework of resource conservation theory, anxiety and depression can be conceptualized as individuals’ physical and mental responses to actual or imagined resource loss. As effective defense mechanisms against resource loss, tenacity and strength (loss oriented resources) enable people to recover from negative experiences and threats (Block and Kremen, 1996). Further research showed that when loss oriented individuals had fewer resources, their coping abilities were compromised, making them unable to effectively face challenging stressors (Demerouti et al., 2004) and exhibiting more physical and mental symptoms. In this study, high strength can significantly reduce the odds of medical students being classified as high anxiety group, and high tenacity and strength can significantly reduce the odds of medical students being classified as high depression group, further verifying the important role of tenacity and strength in resisting resource loss and maintaining physical and mental health. As the saying goes, “there are unpredictable storms in the sky, and misfortunes and blessings in a single day,” in the uncertain risks of modern society, encountering setbacks and difficulties, and bearing possible high pressure, may be a high probability event for every individual, especially for medical students. In this context, improving physical and mental reactions such as anxiety and depression under pressure, strengthening tenacity and strength and other psychological capital, is not only supported by research evidence, but also in line with the value guidance of traditional Chinese culture. For example, emphasizing the cultivation of resilience character such as “good as water” and “resilient as bamboo,” emphasizing the spirit of self-improvement and tolerance by learning from heaven and earth, such as “heaven’s actions are healthy, gentlemen strive for self-improvement; the terrain is vast, and gentlemen carry things with kindness.”

The limitation of this study was that cross-sectional studies could only provide data at a certain time point. Future studies should comprehensively consider longitudinal variation data at different time points to derive more clinically significant symptom subtypes. Secondly, non-random sampling limited the extrapolation of research results, and the universality and representativeness of the results need further verification.

## 5 Conclusion

The research results suggest that there is a considerable degree of stress, anxiety, and depression in the medical student population, and there is significant heterogeneity in their stress, anxiety, and depression manifestations. Specifically, there are three categories of stress latent categories: high stress, low stress, and no stress; two categories of anxiety latent categories: high anxiety, LAGASE; and three categories of depression latent categories: high depression, DGIM, and no depression. Compared with the severity grouping based on norms, the low stress group and DGIM reveal some characteristic behavioral patterns in normal and mildly symptomatic populations.

The three factors of psychological resilience (tenacity, strength, and optimism) have different effects on the latent categories of stress, anxiety, and depression. Optimism only has a significant predictive effect on the latent categories of stress; tenacity and strength have significant predictive effects on the latent categories of depression, and strength only has a significant predictive effect on the latent categories of anxiety.

Future research should combine the severity of stress, anxiety, and depression with their characteristic behavioral patterns, further deepen the study of influencing factors and mechanisms, promote understanding of the complexity and diversity of stress, anxiety, and depression among medical students, and provide more targeted evidence support for psychological intervention for medical students.

## Data Availability

The original contributions presented in the study are included in the article/supplementary material, further inquiries can be directed to the corresponding author.
